# Genotyping of Hydatid Cyst Isolated from Human and Domestic Animals in Ilam Province, Western Iran Using PCR-RFLP

**Published:** 2013

**Authors:** M Dousti, J Abdi, S Bakhtiyari, M Mohebali, SH Mirhendi, MB Rokni

**Affiliations:** 1 Dept. of Medical Parasitology and Mycology, School of Public Health, Tehran University of Medical Sciences, Tehran, Iran; 2 Dept. of Medical Parasitology, School of Medicine, Ilam University of Medical Sciences, Ilam, Iran; 3 Dept. of Medical Biochemistry, School of Medicine, Ilam University of Medical Sciences, Ilam, Iran; 4 Center for Research of Endemic Parasites of Iran (CREPI), Tehran University of Medical Sciences, Tehran, Iran

**Keywords:** *Echinococcus granulosus*, Protoscolices, Genotyping, Strain, Iran

## Abstract

**Background:**

Hydatidosis or cystic hydatid disease is one of the most important diseases in human and animals. Identification of strains is important for improvement of control and prevention of disease. The aim of this study was to determine the strains isolated from human and domestic animals in Ilam Province, Iran, using PCR-RFLP method.

**Methods:**

Respectively, 30 and 4 animal and human hydatid cysts were collected from different slaughterhouses and hospitals of the province. Protoscolices were separated and their DNA genome was extracted by extraction kit. rDNA-ITS1 of each isolated samples was duplicated by BD1(Forward) and 4s (Reverse) Primers. PCR products were studied by electrophoresis and then were digested using TaqI, HpaII, RsaI and AluI restriction enzymes. RFLP products were studied using electrophoresis on 1% agar gel.

**Result:**

A fragment of 1000bp was produced from amplification of rDNA-ITS1 of protoscolices using PCR method. After digestion of PCR product by AluI enzyme, 200bp and 800bp, by RsaI, 655bp and 345bp and by HpaII 700bp and 300bp sizes were obtained. TaqI enzyme had no change in fragment size and it remained 1000bp. Considering the method, Ilam strains was specified as *E. granulosus* sensu stricto (G1-G3).

**Conclusions:**

Although sheep strain (G1) is dominated in human and different animal in Iran and the world, but more efforts should be done to clarify the true genotype of Ilam strains specified as *E. granulosus* sensu stricto (G1-G3).

## Introduction

Hydatidosis is one of the most important diseases in human and animals distributed in many countries all over the world and in Iran too. The disease caused by intestinal cestodes of dogs named *Echinococcus granulosus*. Humans are affected by ingesting contaminated vegetables, food or water. The disease is important not only in human, due its affect on critical organs such as liver and lung, but in livestock because of remarkable economical damage in stock breeding (1, 2).

Iran is endemic region for hydatidosis and about 1% of surgeries is related to it, so exact studies of this disease from different aspect is necessary (3). Different studies have been conducted to detect the prevalence of the disease in various provinces of Iran. A rate of 1.2-21.4% of seropositivity is obvious in different parts of the country (3, 4). Echinococcosis in dogs is reported from 2.2% to 48% in different regions of Iran (3). Several slaughterhouse based studies shows that hydatid cyst infection is from 1.5% to 70% in animals (3, 5). The main cycles for parasite transmission are dog-sheep cycle in Iran. The final hosts in Iran besides dogs are jackal and wolf (6–8). In addition, besides sheep; cow, camel, goat and buffalo are intermediate host (9).

Not mention of previous studies including different aspects such as epidemiology, prevalence, incidence, diagnosis, etc, it sounds obligatory to appraisal the genotype of *E. granolusus* in affected parts of Iran. Ilam Province, western Iran, due to its climatic conditions, high population, and its tribes which keep dog for their stockbreeding purposes is considered a high risk region for hydatidosis (10). Usually in some regions of Iran *E. granulosus* has various genetics with different genotype complex (11). Overall 10 genotypes have been identified for this parasite (G1-G10) (1, 5, 12, 13). Genotype diversity and *E. granulosus* complex have been shown that can affect on hydatidosis evolution, transitions dynamics, making illness, antigenicity, immunization, drugs reactions, epidemiology and disease control (12, 13).

The aim of this study was to identify strains of hydatid cyst in human and animals in Ilam Province, western Iran.

## Materials and Methods

**Sample collection:** Totally, 34 hydatid cysts including 30 hydatid cysts of sheep and cow from Ilam slaughterhouse and 4 human hydatid cysts from Ilam hospitals were collected and sent to Parasitology Laboratory of Ilam University of Medical Sciences. Protoscolices were separated under sterile conditions. The surface of cysts was washed by cotton and 70% alcohol and protoscolices and liquid were extracted from cysts. Protoscolices were separated by centrifuging and stored in alcohol 70% until use.

**Genetics studies:** This step included three stages as follows: DNA extraction, PCR and digestion (RFLP).

### DNA extraction

At first, protoscolices were washed with distilled water three times, and then genomic DNA from samples was extracted by extraction kit (Bioneer Company, South Korea).

### PCR

PCR were performed as described earlier (11, 14) with some modification. Using PCR, a sequence of 1000bp from rDNA-ITS1 fragment was amplified by Forward (5- GTC GTA ACA AGG TTT CCG TAG G-3) and Reverse primers (5- TAG CGT TCG AAG TGT CG-3) manufactured by Sinagene Company (Tehran, Iran). These primers were used in the previous researches (11). Total volume of 20 µl was selected for PCR reaction (Taq DNA polymerase one unit, MgCl2 1 µl, forward and Reverse primers 2 µl, DNA 1 µl and buffer 10X 2 µl, dNTP one µl and distilled water 13.5 µl). After preparing PCR mixture, thermocycler program was regulated on 35 cycles for DNA duplication as follows: The primary denaturation step in 95 °C 3 min, denaturation step in 95 °C 1 min, annealing step in 55 °C 45 seconds, extention step in 72 °C 1 min and 20 second (steps 2-4 were replicated 35 cycles) and final extention step in 72 °C 10 min, then the product of PCR was electrophoresed on 1% agar gel.

### Digestion

For enzymatic digestion of PCR products, enzymes AluI, RsaI, HpaII and TaqI, which identify sequences of AG/CT, GT/AC, C/CGG and T/CGA respectively, were used (11, 15, 16). Enzyme digestion was done in total reaction of 20 µl including 2 µl buffer, 10 µl PCR products and 1 µl (10 units) enzyme up to 20 µl by distilled water. At the next step, the content of digestion reaction contains AluI, HpaII, and RsaI was incubated at 37 °C for 12 h (by suggestion of enzyme Producer Company). This temperature was 65 °C for TaqI enzyme. After passing incubation times, enzyme digestion product was eletrophoresed on 1% agar gel and then the created bands were photographed and observed by documentation gel system.

## Results

**Fig. 1:**
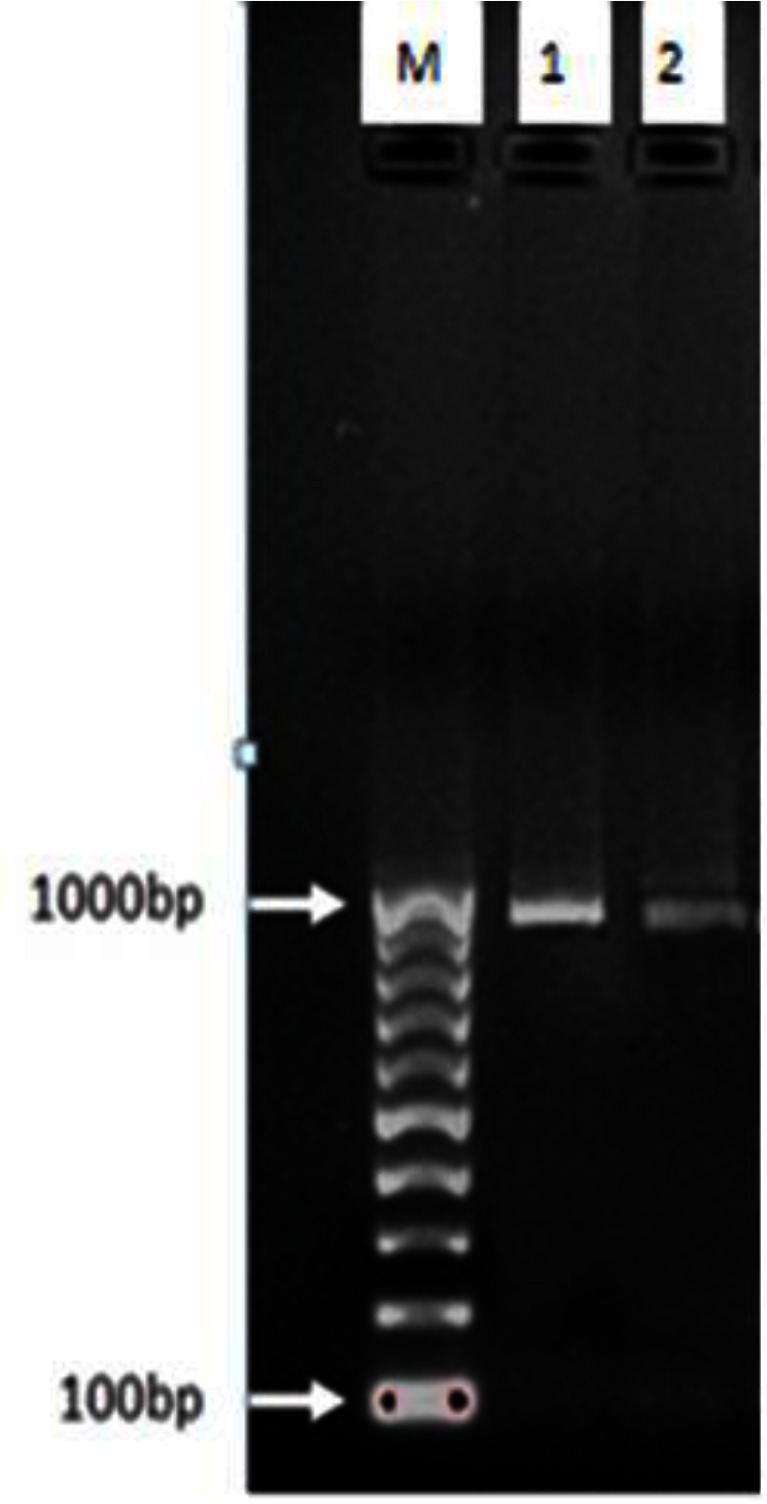
PCR products of rDNA-ITS1 of protoscolices on agar gel: m: marker with 100bp molecular weight, 1&2: Sample

**Fig. 2:**
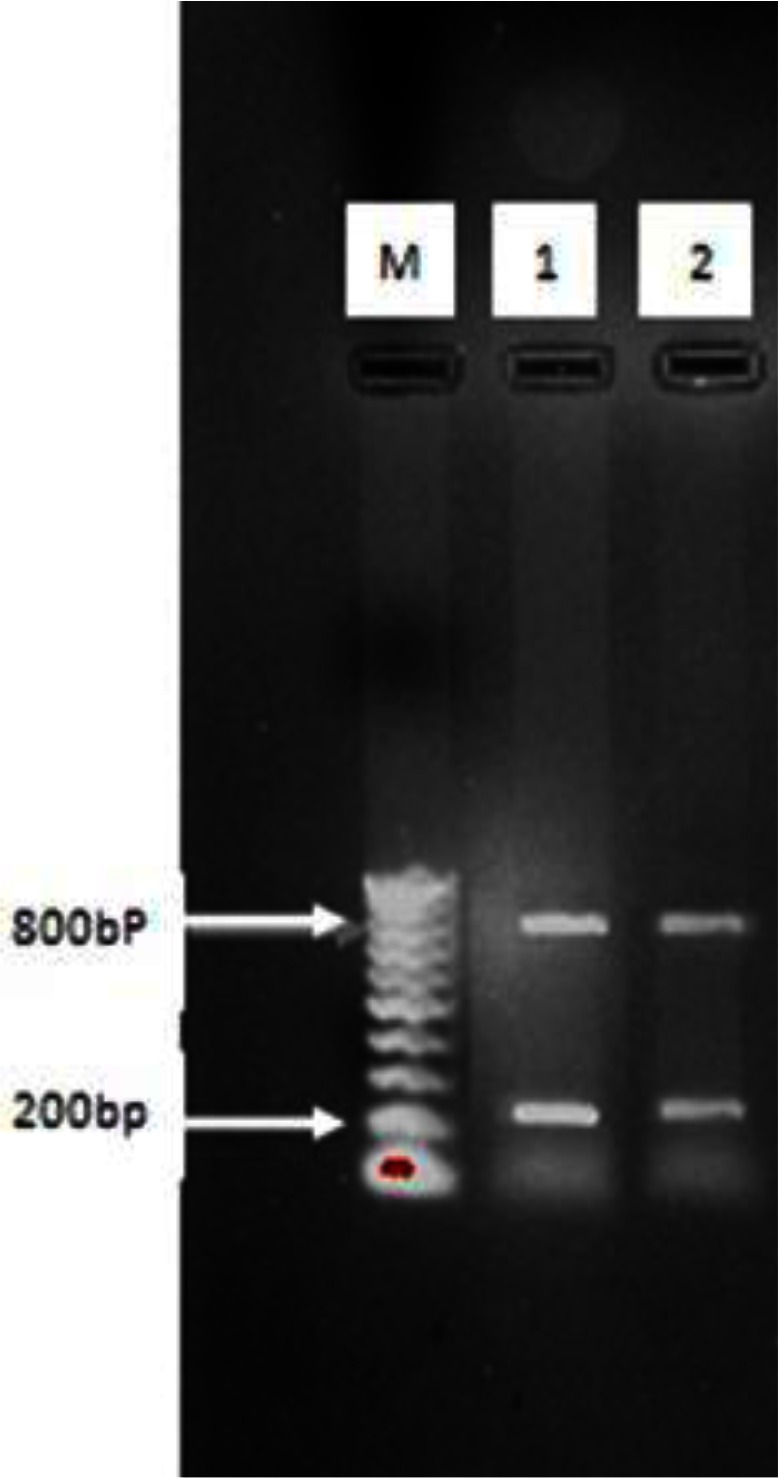
Digestion pattern of 1000bp PCR products of rDNA-ITS1 fragment with AluI enzyme on agar gel: M: marker with 100bp molecular weight, 1, 2: samples

**Fig. 3:**
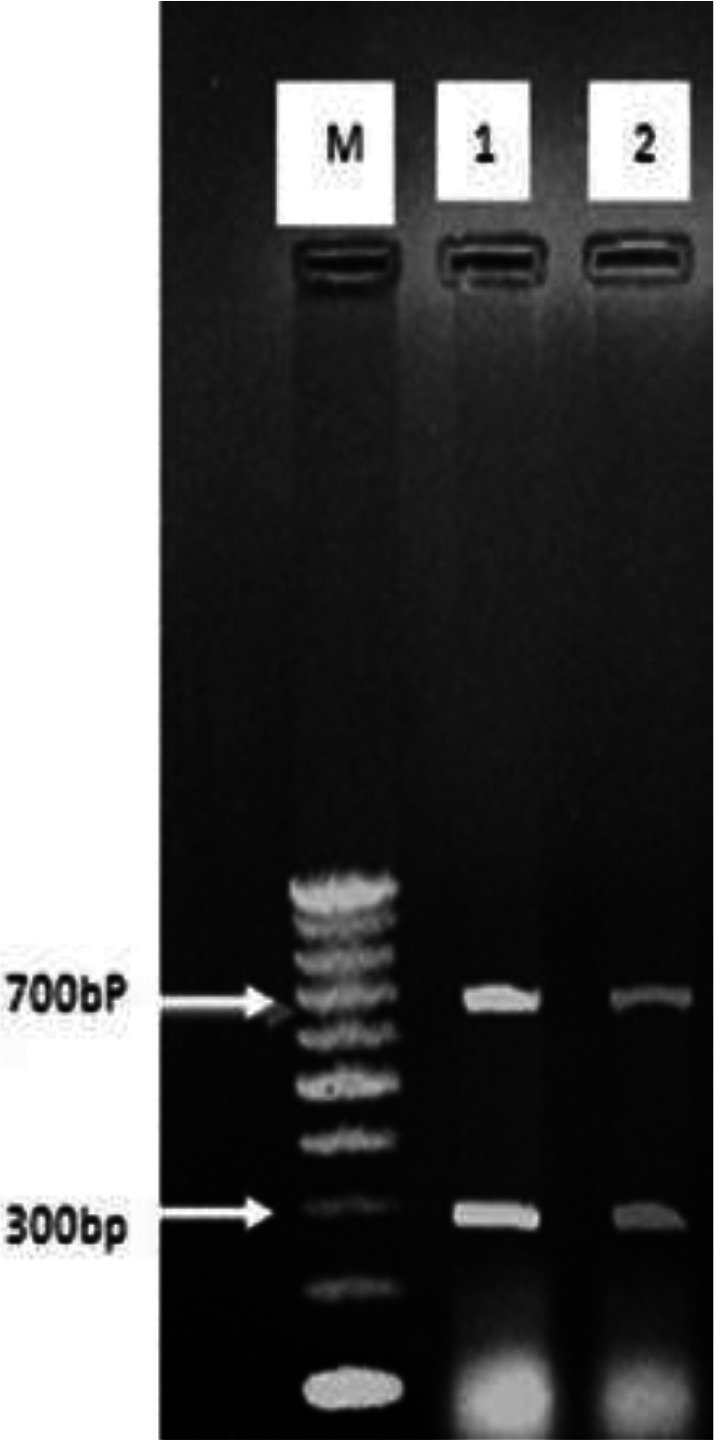
Digestion pattern of 1000bp PCR products from rDNA-ITS1 fragment with HpaII enzyme on 1% agar gel: M: marker with 100bp molecular weight, 1, 2: samples

**Fig. 4:**
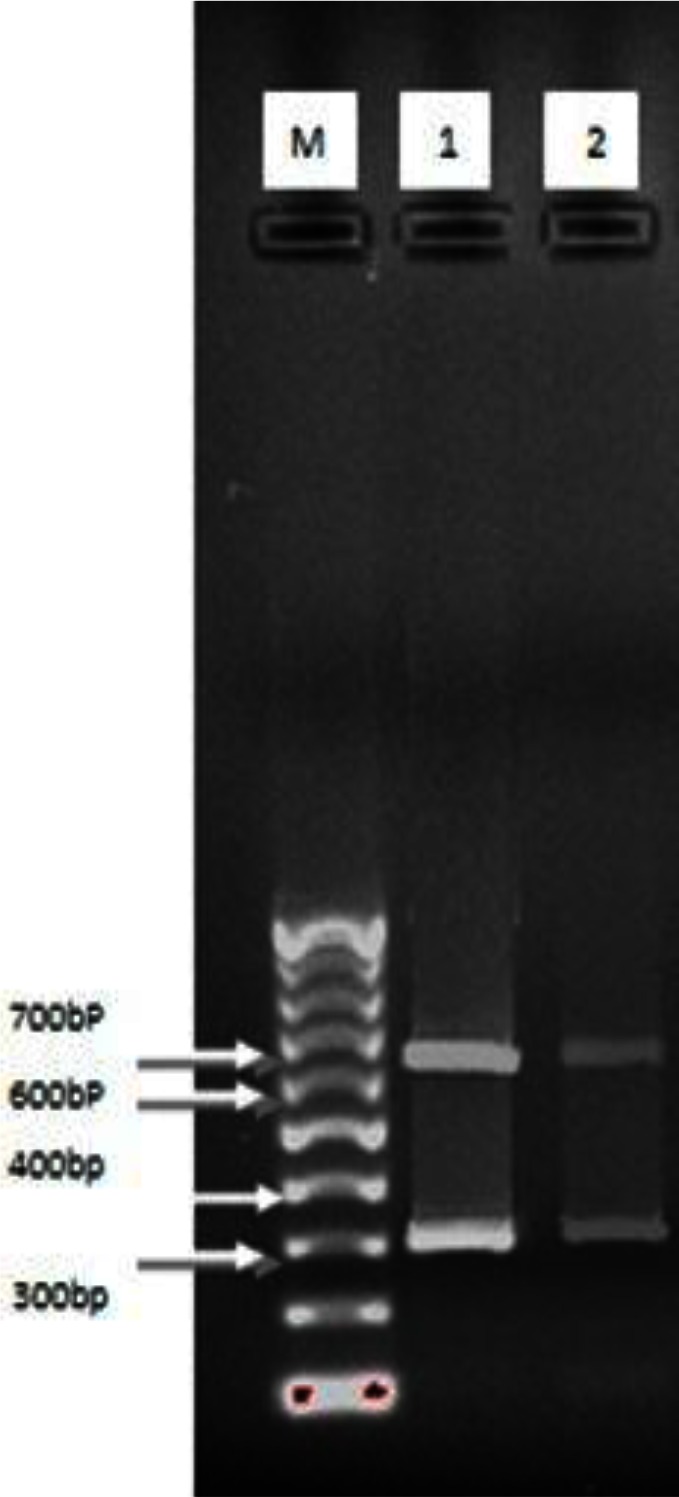
Digestion pattern of 1000bp PCR products from rDNA-ITS1 fragment with RsaI enzyme on agar gel: M: marker with 100bp molecular weight, 1,2: samples

**Fig. 5:**
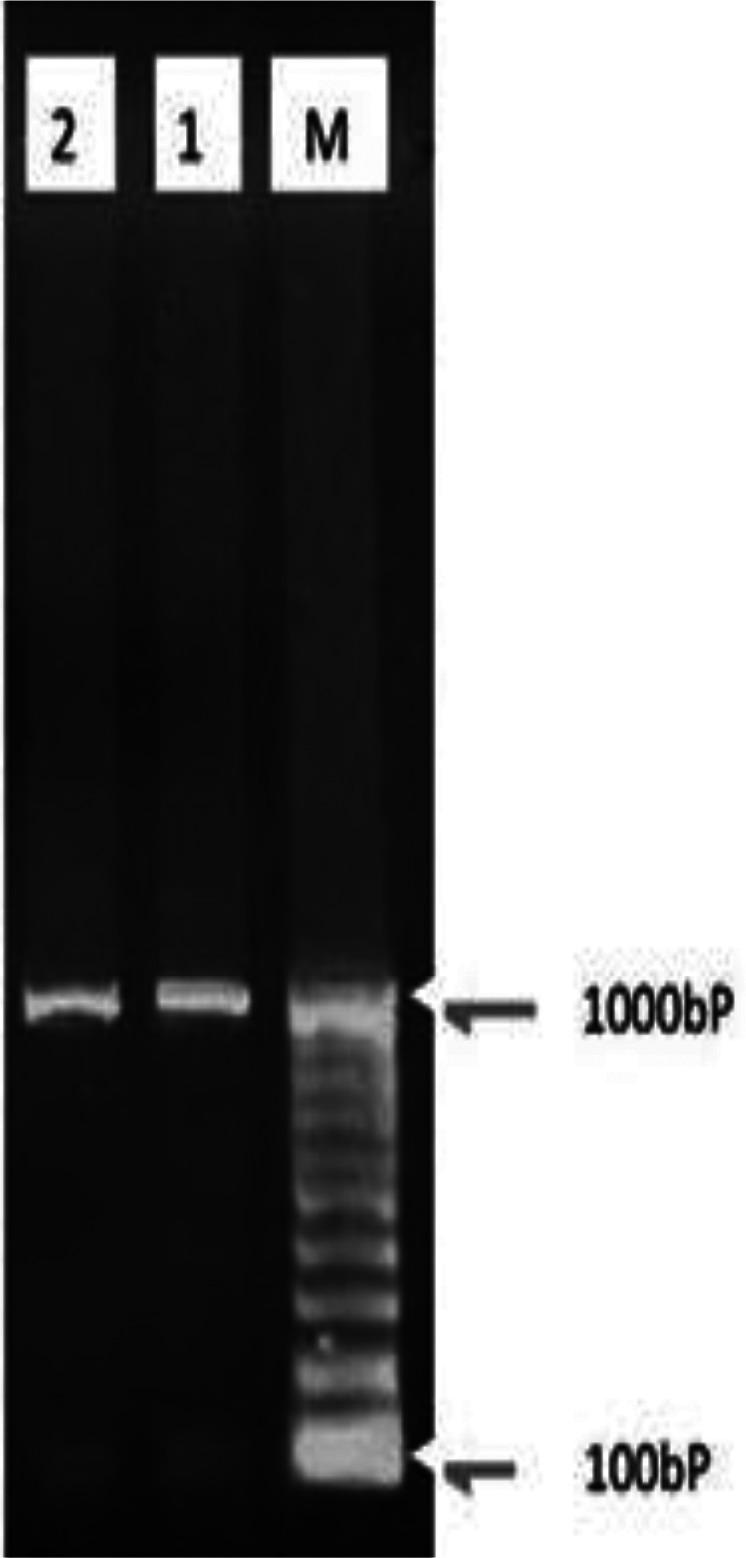
Digestion pattern of 1000bp PCR products from rDNA-ITS1 fragment with TaqI enzyme on agar gel: M: marker with 100bp molecular weight, Column 1, 2: samples

rDNA-ITS1 fragment of 32 samples including 20 from sheep (14 livers and 6 lungs), 10 from cow (6 livers and 4 lungs) and 2 human origin (liver), was amplified by polymerase chain reaction. The length of amplified fragment for all isolated samples with sheep, bovine and human origin was 1000bp (Fig. 1). This fragment was digested by enzymes and with ALuI restriction enzyme yielded 200 and 800 bp fragments (Fig. 2); with HpaII, 300 and 700 bp fragments (Fig. 3); and RsaI, 345 and 655 bp fragments (Fig. 4). The TaqI restriction enzyme had no effect on PCR product and after digestion intact 1000bp fragment was seen (Fig. 5). Results of this study demonstrated that dominant strain of *E. granulosus* in Ilam was *E. granulosus* sensu stricto (G1-G3).

## Discussion

This research was conducted to identify and determine *E. granulosus* larval stage genotype in Ilam, western Iran in order to complete the parasite's gene map in Iran and valuable information was obtained. In this research rDNA-ITS1 fragment was amplified by PCR and PCR products were eletrophoresed. 1000bp bands were observed from amplification of rDNA-ITS1 in all samples. After digestion of PCR products by restriction enzymes, obtained fragments demonstrated as *E. granulosus* sensu stricto (G1-G3).

Several studies have been conducted on genotyping of *E. granulosus* in Iran. Yoosefi, et al., using PCR-RFLP method, reported that dominant strain of *E. granulosus* in Chaharmahal- va -Bakhtyari, central Iran, was G1 (17). A similar study in Isfahan Province, central Iran, on hydatid cysts with origination of cow, sheep, goat, camel and human, showed G1 as the dominant strain (18). Studying on 16 hydatid cyst isolated from Iranian patients and several domesticated animals such as cow, sheep and camels in Tehran, by Zhang, et al. (19) showed that camel had G6 genotype, sheep had G1 genotype and human had G1 genotype. Yakhchali, et al. (20) reported that all samples isolated in definitive and intermediate hosts of the parasites belonged to G1 genotype (sheep strain). Sadri, et al. (21) by studying 93 isolated hydatid cysts from slaughtered livestock of Yasuj City, showed that all samples were G1 genotype (sheep strain). These findings are more or less in accordance with our research results.

Sharbatkhori, et al. (23) by sequencing analysis of mitochondrial cytochrome c oxidase subunit 1 (cox1) and NADH dehydrogenase subunit 1 (nad1) genes, on 19 hydatid cyst isolates collected from camels in central Iran, demonstrated the presence of G3 genotype (buffalo strain) of *E. granulosus* as dominant genotype in camels. Besides, Pezeshki, et al. (22) by studying 55 isolated hydatid cysts from domestic animals and humans from Ardabil Province, showed G1 as the dominant strain and for the first time in Iran reported G3 genotype in 2 Human isolates. Forthcoming notes in this paper will show that it is possible that some isolates from Ilam be detected as G3 genotype.

Outside Iran studies have also been conducted about genotyping of hydatid cyst. A study on 91 hydatid cysts isolated from domestics animal of Sardinia Island by PCR and PCR-RFLP for determining genotype of *E. granulosus* showed that 89 samples of 91 samples in sheep, cow and pig belonged to G1 genotype but hydatid cyst of two pigs was G7 genotype (24). This study is also in line with our results.

The study encompassed two limitations. The first is the small number of human samples. The reality is that due to the high risk of surgery in this disease many people prefer to refer to more developed hospitals, whether Tehran or nearby provinces, for this purpose. This issue prohibited the authors to collect more human samples so in interpretation of the results caution should be taken.

The second is that PCR-RFLP is not capable to differentiate G1-G3 from each other, so to better specify the true genotype of our strains they should be examined via gene sequencing using nad1 and cox1, as some previous studies conducted in Iran had utilized it. Considering the aims of the study and restriction of financial sources until complementary studies, we have no choice but to specify Ilam strains as *E. granulosus* sensu stricto (G1-G3) proposed by Mc Manus and Thompson (25).

## Conclusion

This study showed that the main strain for *E. granulosus* in Ilam Province was *E. granulosus* sensu stricto (G1-G3) similar to other provinces in Iran. The finding might be considered as a complementary part of the puzzle on determining the gene map of the parasite in Iran. Further studies should be conducted to be sure of the sheep strain G1 which is predominant strain in Iran.
